# Supplementary material for: Dynamic Interplay of Metabolic and Transcriptional Responses in Shrimp during Early and Late Infection Stages of Enterocytozoon hepatopenaei (EHP)
Source: Int J Mol Sci. 2023 Nov 25;24(23):16738. doi: 10.3390/ijms242316738 (PMC10706788; doi:10.3390/ijms242316738)
Supplement: Supplementary file 1 [file ijms-24-16738-s001.zip › Figure legends.pdf]

Figure S1. The distribution of reads mapped to the genome on different chromosomes. The x-axis represents different chromosomes, and the y-axis represents the number of aligned reads on the chromosome.

Figure S2. Venn diagrams for the four groups. Different colors indicate four different groups. The numbers in the overlapping part represent the numbers of DEGs shared between groups, while the numbers in non-overlapping part represent the numbers of DEGs unique to each group.

Figure S3. Comparison of expression of twenty-four genes measured by RNA-Seq and qRT-PCR. (a): Asn vs Asp, (b): Jsn vs Jsp, (c): Asn vs Jsn, (d): Asp vs Jsp. 18s RNA is the internal reference gene control, blue bars indicate qRT-PCR, and yellow bars indicate RNA-Seq. Data shown are the mean of triplicate ± SD.

Figure S4. The p value histogram of KEGG enrichment analysis for integrated metabolomics and transcriptomics. (**a**): Asn vs Asp, (**b**): Jsn vs Jsp. The x-axis represents the enrichment factor, and the y-axis is the KEGG pathway name. The closer to red, the more significant the p value is.

Figure S5. Boxplot of 18s RNA Ct Values Across Comparative Groups. The boxplot illustrates the distribution of Ct values for the internal reference gene (18s RNA) across four comparative groups. Each box represents the interquartile range (IQR) of Ct values within a group, with the horizontal line inside the box denoting the median. (Group **B - H vs E** represents the distribution of CT values when we used the same internal reference gene.)
