# Supplementary material for: Dynamic Interplay of Metabolic and Transcriptional Responses in Shrimp during Early and Late Infection Stages of Enterocytozoon hepatopenaei (EHP)
Source: Int J Mol Sci. 2023 Nov 25;24(23):16738. doi: 10.3390/ijms242316738 (PMC10706788; doi:10.3390/ijms242316738)
Supplement: Supplementary file 1 [file ijms-24-16738-s001.zip › Figure S4.pdf]

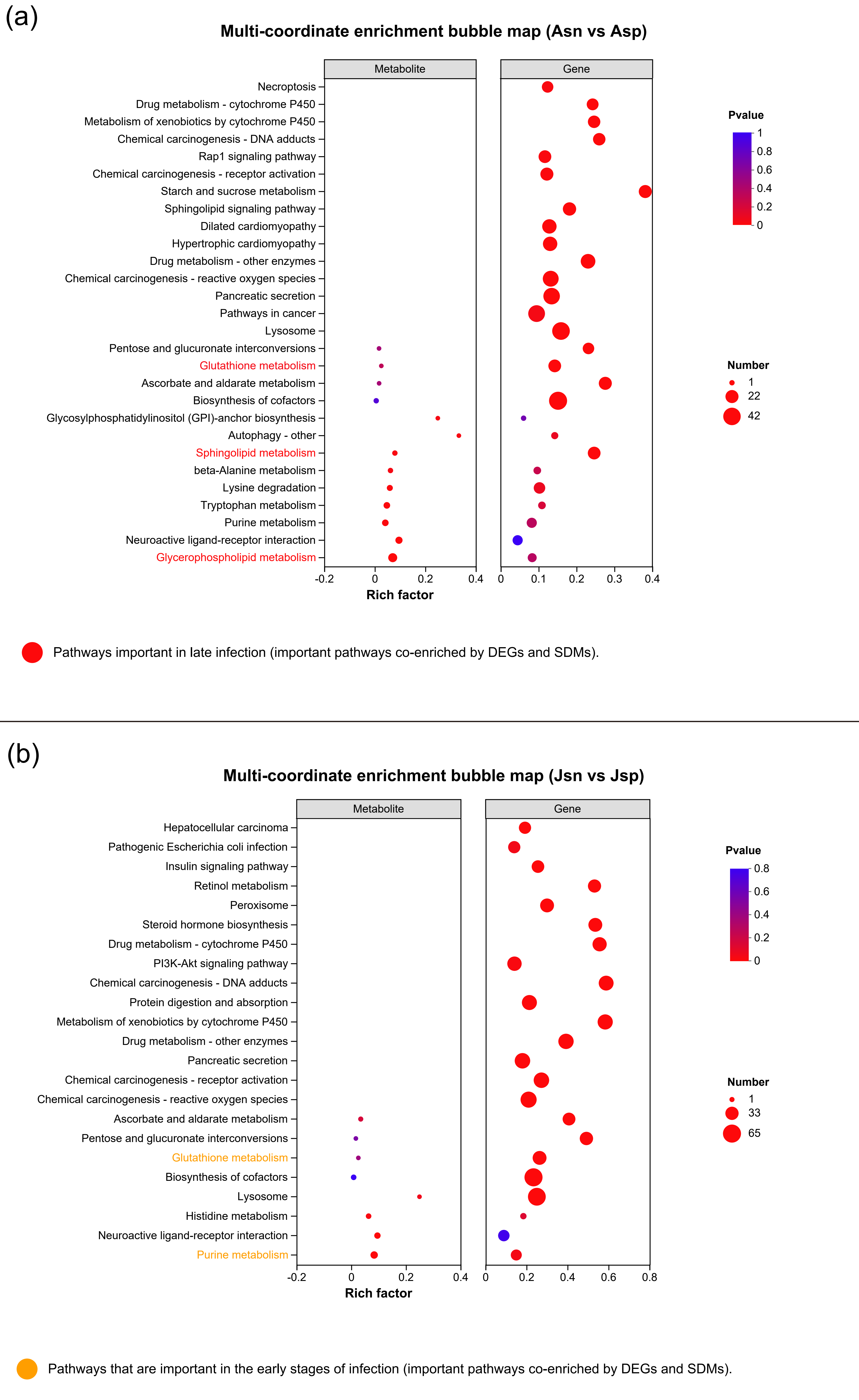
(a)
Multi-coordinate enrichment bubble map (Asn vs Asp)
Metabolite
Gene
Necroptosis
Drug metabolism - cytochrome P450
Metabolism of xenobiotics by cytochrome P450
Chemical carcinogenesis - DNA adducts
Rap1 signaling pathway
Chemical carcinogenesis - receptor activation
Starch and sucrose metabolism
Sphingolipid signaling pathway
Dilated cardiomyopathy
Hypertrophic cardiomyopathy
Drug metabolism - other enzymes
Chemical carcinogenesis - reactive oxygen species
Pancreatic secretion
Pathways in cancer
Lysosome
Pentose and glucuronate interconversions
Glutathione metabolism
Ascorbate and aldarate metabolism
Biosynthesis of cofactors
Glycosylphosphatidylinositol (GPI)-anchor biosynthesis
Autophagy - other
Sphingolipid metabolism
beta-Alanine metabolism
Lysine degradation
Tryptophan metabolism
Purine metabolism
Neuroactive ligand-receptor interaction
Glycerophospholipid metabolism
-0.2
0
0.2
0.4
0
0.1
0.2
0.3
0.4
Rich factor
Pvalue
1
0.8
0.6
0.4
0.2
0
Number
1
22
42
Pathways important in late infection (important pathways co-enriched by DEGs and SDMs).
(b)
Multi-coordinate enrichment bubble map (Jsn vs Jsp)
Metabolite
Gene
Hepatocellular carcinoma
Pathogenic Escherichia coli infection
Insulin signaling pathway
Retinol metabolism
Peroxisome
Steroid hormone biosynthesis
Drug metabolism - cytochrome P450
PI3K-Akt signaling pathway
Chemical carcinogenesis - DNA adducts
Protein digestion and absorption
Metabolism of xenobiotics by cytochrome P450
Drug metabolism - other enzymes
Pancreatic secretion
Chemical carcinogenesis - receptor activation
Chemical carcinogenesis - reactive oxygen species
Ascorbate and aldarate metabolism
Pentose and glucuronate interconversions
Glutathione metabolism
Biosynthesis of cofactors
Lysosome
Histidine metabolism
Neuroactive ligand-receptor interaction
Purine metabolism
-0.2
0
0.2
0.4
0
0.2
0.4
0.6
0.8
Rich factor
Pvalue
0.8
0.6
0.4
0.2
0
Number
1
33
65
Pathways that are important in the early stages of infection (important pathways co-enriched by DEGs and SDMs).
