# Supplementary figures and images for: Dynamic Interplay of Metabolic and Transcriptional Responses in Shrimp during Early and Late Infection Stages of Enterocytozoon hepatopenaei (EHP)
Source: Int J Mol Sci. 2023 Nov 25;24(23):16738. doi: 10.3390/ijms242316738 (PMC10706788; doi:10.3390/ijms242316738)

## Reads distribution in chromosomes

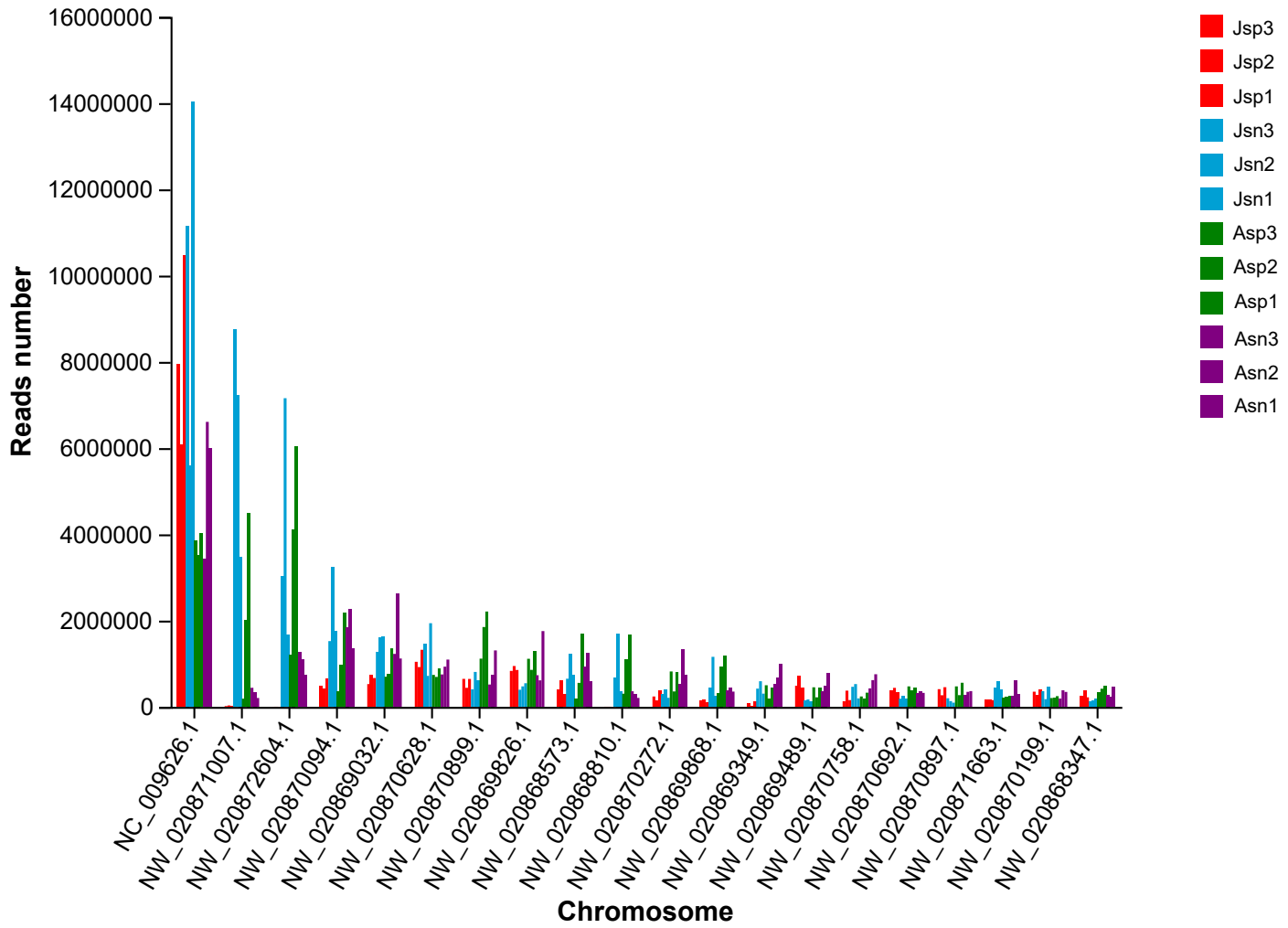

Supplement: Supplementary file 1 [file ijms-24-16738-s001.zip › Figure S1.pdf]

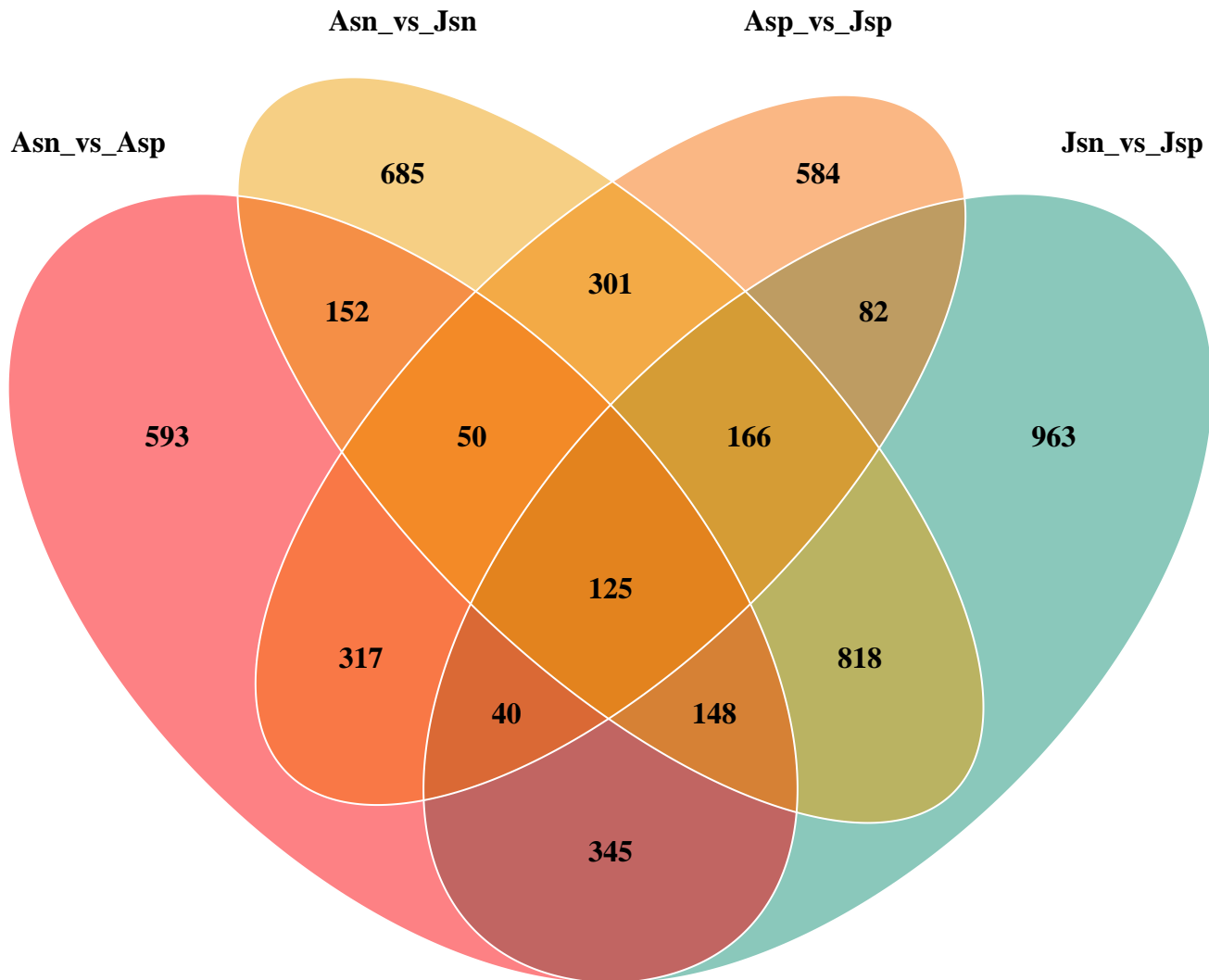

Supplement: Supplementary file 1 [file ijms-24-16738-s001.zip › Figure S2.pdf]

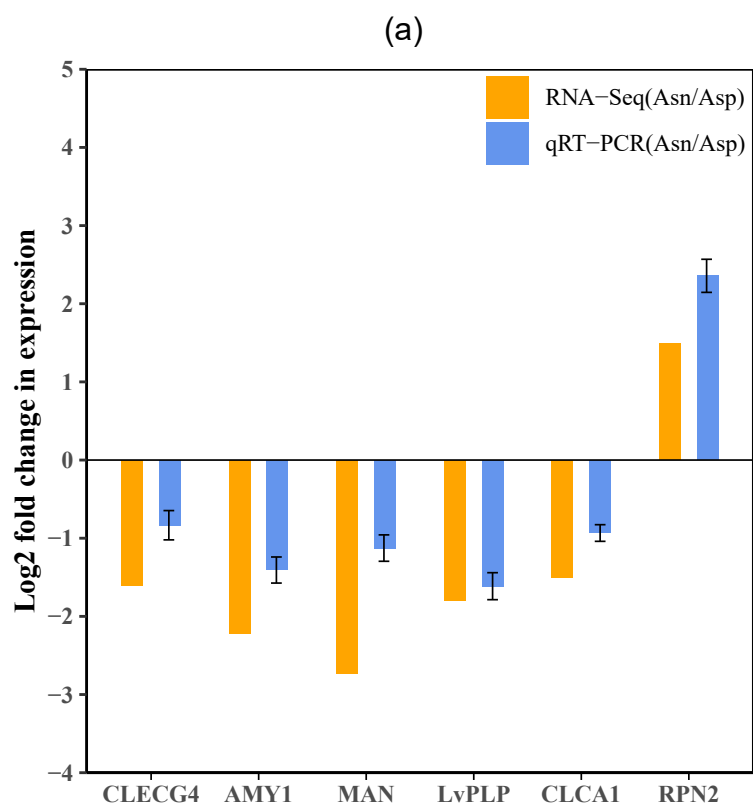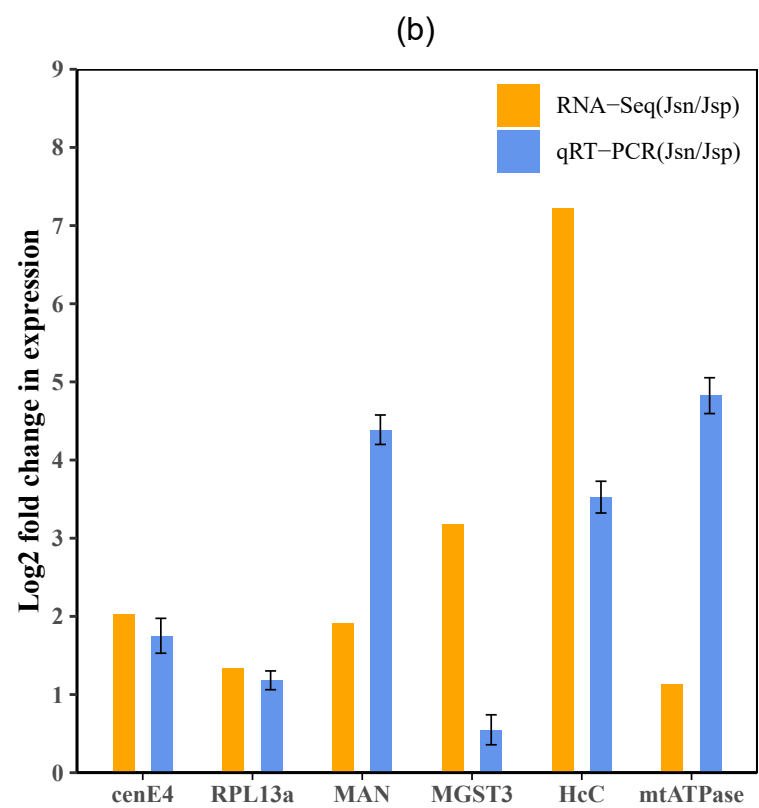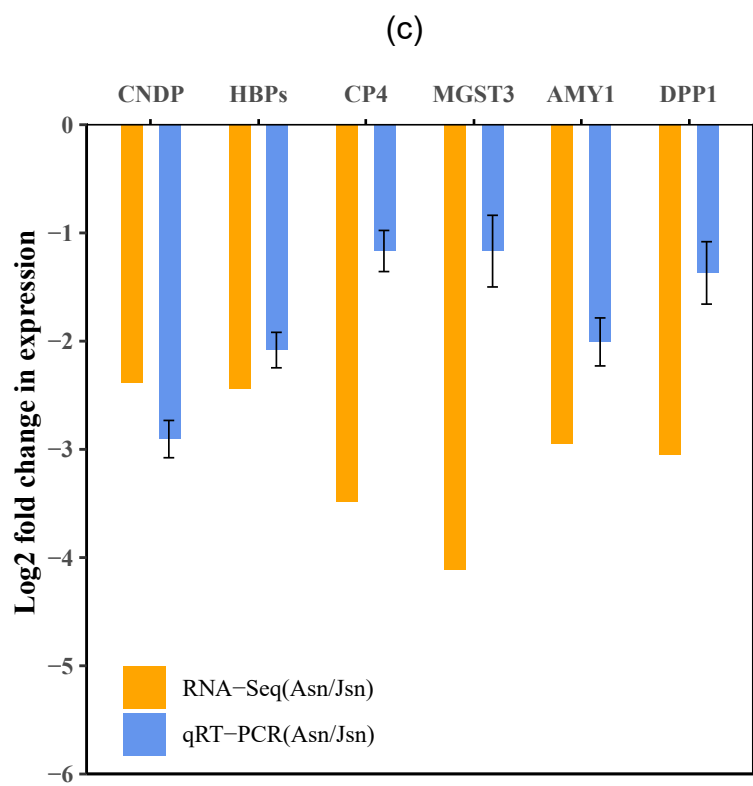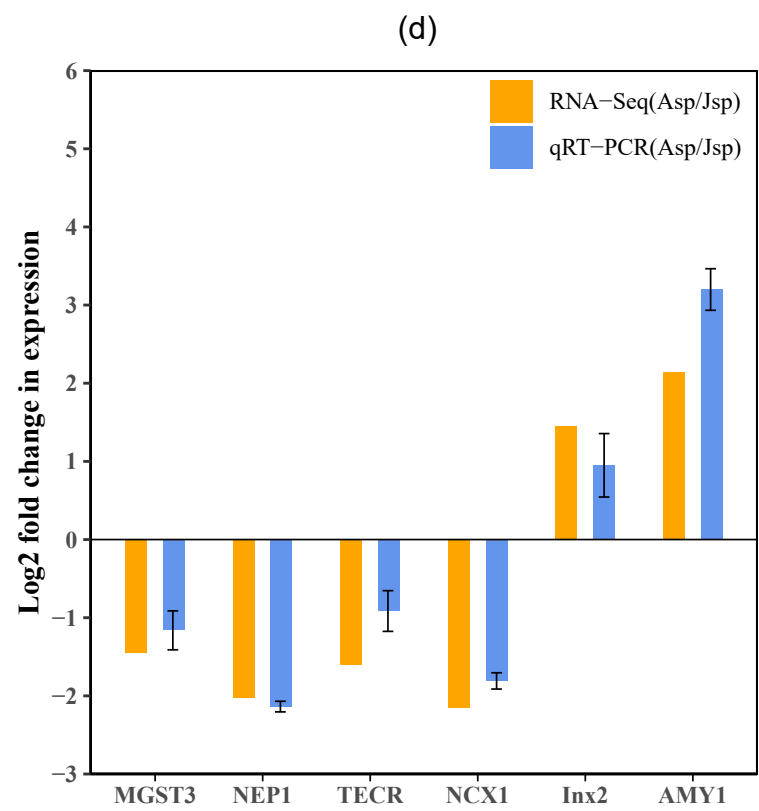

Supplement: Supplementary file 1 [file ijms-24-16738-s001.zip › Figure S3.pdf]

**Boxplot of 18s RNA Ct Values Across Groups**

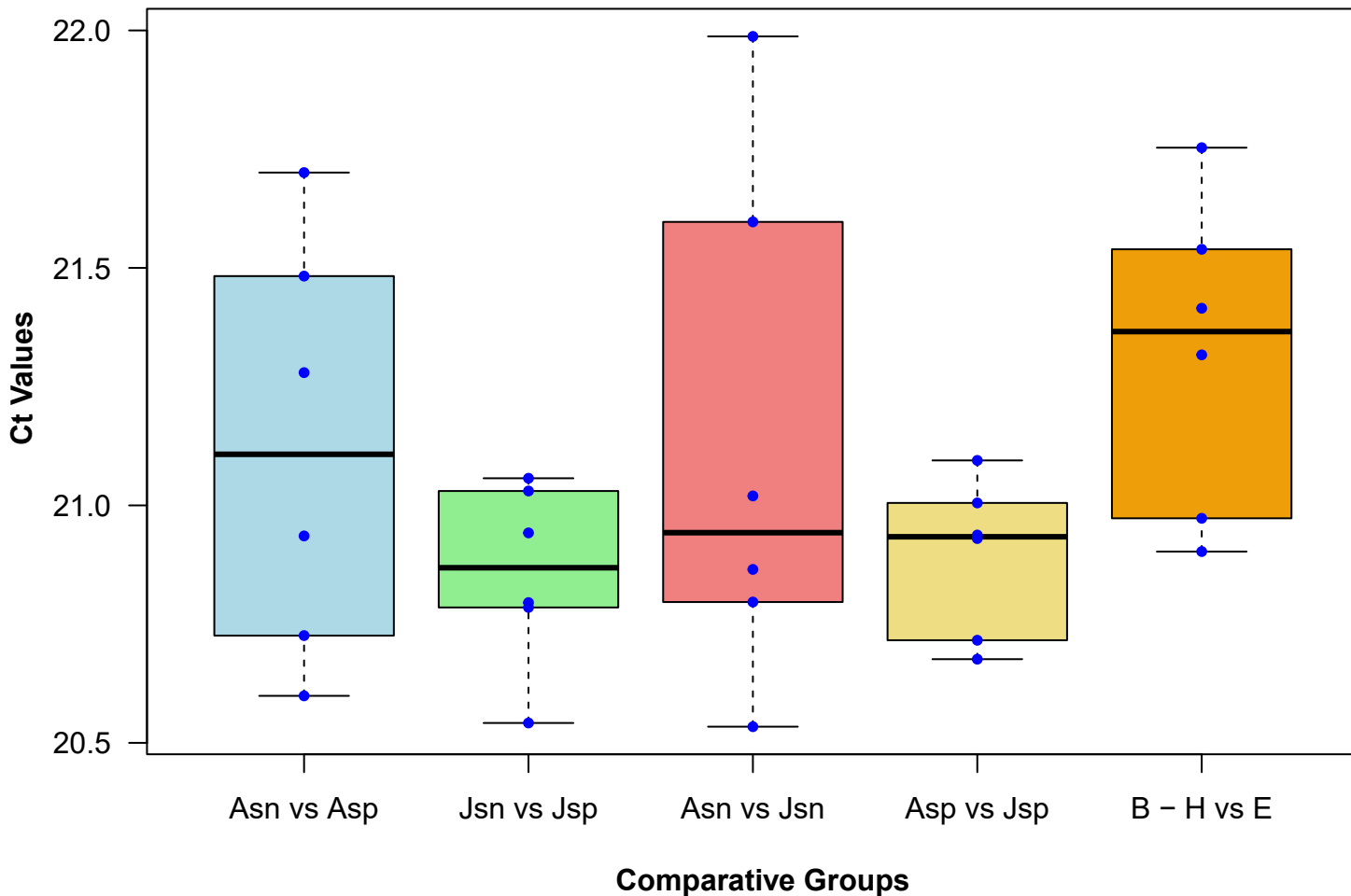

Supplement: Supplementary file 1 [file ijms-24-16738-s001.zip › Figure S5.pdf]
